# Maackiain Prevents Amyloid-Beta–Induced Cellular Injury via Priming PKC-Nrf2 Pathway

**DOI:** 10.1155/2022/4243210

**Published:** 2022-06-22

**Authors:** Na Lu, Guojun Tan, Hongling Tan, Xing Zhang, Yunling Lv, Xiujuan Song, Daofeng You, Ziyuan Gao

**Affiliations:** ^1^Department of Emergency Intensive Care Medicine, The First Hospital of Hebei Medical University, Shijiazhuang, China; ^2^Department of Neurology, The Second Hospital of Hebei Medical University, Shijiazhuang, China; ^3^Department of Gastrointestinal Surgery, The First Hospital of Hebei Medical University, Shijiazhuang, China; ^4^Department of Emergency Intensive Care Medicine, The Second Hospital of Shijiazhuang City, Shijiazhuang, China

## Abstract

Amyloid-beta (A*β*) peptide induces neurotoxicity through oxidative stress and inflammatory response. Brain deposition of a large amount of amyloid-beta (A*β*), in particular A*β*_42_, promotes the development of Alzheimer's disease (AD). Maackiain is extracted from traditional Chinese medicine peony root and possesses antioxidative, antiosteoporosis, antitumor, and immunoregulatory effects. Whether Maackiain can reduce neurotoxicity caused by A*β* accumulation remains elusive. Herein, we found that Maackiain downregulated A*β*_42_-induced cell injury and apoptosis in PC12 cells. Moreover, Maackiain prevented A*β*_42_ stimulation-induced generation of oxidative stress and reduced A*β*_42_-caused impairment of mitochondrial membrane potential in PC12 cells. Maackiain increased the superoxide dismutase activity and decreased malondialdehyde content that was induced by A*β*_42_. Mechanistic studies showed that Maackiain increased intranuclear Nrf2 expression. Consistently, Nrf2 silencing by RNA interference weakened the protective role of Maackiain against A*β* exposure. In addition, calphostin C, a specific antagonist of protein kinase C, attenuated the promoting effects of Maackiain on Nrf2 nuclear translocation. Moreover, calphostin C attenuated the antioxidant and anti-inflammatory capabilities of Maackiain in PC12 cells. Collectively, Maackiain promoted Nrf2 activation through the PKC signaling pathway, thus preventing PC12 cells from A*β*-induced oxidative stress and cell injury, suggesting that Maackiain is a potential drug for AD treatment.

## 1. Introduction

Alzheimer's disease (AD) is the most common neurodegenerative disease and is characterized by senile plaques, neurofibrillary tangles, and loss of nerve cells and synapses [1]. Amyloid-beta (A*β*) especially A*β*_42_ is the main component of senile plaques and promotes the formation of neurofibrillary tangles and loss of synapses during the progression of AD leading to neuronal apoptosis [2, 3]. Oxidative stress and inflammatory response have been shown to contribute to A*β*_42_-induced neurotoxicity. Deposition of A*β*_42_ in the brain decreases mitochondrial redox activity and induces the generation of a mass of reactive oxygen species (ROS), leading to the occurrence of oxidative stress in the nervous system [4, 5]. Excessive oxidative stress reaction induces neural inflammatory response through multiple signaling pathways such as nuclear factor kappa B (NF-*κ*B) pathways, thereby further worsening nervous system injury [4, 6, 7]. Therefore, prevention of oxidative stress and neuroinflammatory response is a potential approach for the development of AD neuroprotective drugs.

NF-E2-related factor 2 (Nrf2) functions as a pivotal transcription factor that modulates oxidative stress reaction. Nrf2 is sequestered in cytoplasm under physiological condition by direct binding to Kelch-like ECH-associated protein 1 (Keap1), which prevents the translocation and activity of Nrf2 [8]. In response to internal and external environment stress, such as increase of free oxygen radicals, Nrf2 is liberated from Keap1-Nrf2 complex and translocated into the nucleus, where it promotes the transcription of antioxidant genes [8]. Nrf2 plays an essential role in maintenance of the physiological states of the brain. Nrf2 knockout mice show proteasomal dysfunction and apoptosis in neuron, as well as age-related atrophy of the basal forebrain and neurobehavioral impairment [9, 10]. Nrf2 deregulation is strongly linked to the pathophysiology of AD. Nrf2 expression as well as its inactivation is decreased in the brain of AD patients [11]. In A*β* deposition-related APP/PS1 mice hippocampal Nrf2 expression is decreased [12]; however, injection of lentiviral vectors overexpressing Nrf2 into hippocampus remarkably increases the cognitive and learning abilities of the APP/PS1 mice [13]. In addition, the therapeutic effects of some antioxidants on APP/PS1 mice associated with Nrf2 activation [14–16]. Importantly Nrf2 has been demonstrated to prevent against A*β*-induced oxidative stress reaction and reduce inflammation during the pathological progression of AD [17]. Therefore, Nrf2-targeting drugs are promising in the clinical treatment of AD.

Maackiain, a typical isoflavonoid, is extracted from traditional Chinese medicine peony root. Maackiain possesses antioxidative [18], antiseptic [19], antitumor [20], and immunoregulatory properties [21]. Maackiain has recently been reported to exhibit beneficial effects on preventing and improving diabetes mellitus-related metabolic disturbance [18]. Moreover, Maackiain can reduce dopaminergic neuron damage and improve neurological deficits of *Caenorhabditis elegans* with Parkinson's disease [22]. However, it remains to be explored whether Maackiain can alleviate A*β*-induced neurotoxicity. In the study, we investigated the therapeutic properties of Maackiain in treatment of oxidative stress and inflammation in PC12 cells exposed to A*β* and the underlying mechanisms. Our results demonstrate that Maackiain protects PC12 cells against A*β* exposure through Nrf2 activation in a PKC signaling pathway-dependent manner. Our findings highlight that Maackiain can provide a potential avenue for clinical treatment of AD.

## 2. Materials and Methods

### 2.1. Cell Culture

PC12 cells derived from rat pheochromocytoma were purchased from ATCC and cultured in RMPI 1640 culture medium (Gibco, Carlsbad, CA, USA) containing 10% fetal bovine serum at 37°C in a 5% CO_2_ incubator. Culture medium was refreshed once every 3 days. A*β*_42_ (Abcam, cat # ab120301, USA) was dissolved in dimethyl sulfoxide (DMSO) to a final concentration of 1 mM and then incubated at 37°C for 4 days. Maackiain (Sigma-Aldrich) was dissolved into dimethyl sulfoxide to a concentration of 10 mM. Fresh PC12 cell culture medium was added 6 h prior to A*β*_42_ stimulation.

### 2.2. siRNA Transfection

Nrf2 and control siRNAs were transfected into PC12 cells in the presence of Lipofectamine 3000 (Invitrogen) according to the protocol provided by the manufacturer. Briefly, PC12 cells were inoculated into a 6-well plate and transfected with 50 nM siRNA when reaching 70-80% confluency.

### 2.3. Cell Counting Kit-8 (CCK-8) Assay

Cell viability of the PC12 cells was determined by CCK-8 assay as previously described [23]. Briefly, the cells were inoculated into a 96-well plate (2000 cells/well). After Maackiain treatment with or without A*β* exposure, CCK-8 solution (Sigma-Aldrich) was added into the medium, and the cells were incubated for additional 2 h. Absorbance at 450 nm was determined using a microplate reader (CANY, Shanghai, China).

### 2.4. Western Blot Analysis

Total cell protein was extracted by a Tris lysis buffer (50 mM Tris-base, 150 mM sodium chloride) with 1% Triton. The protein levels were determined by immunoblotting using specific antibodies according to a previous standard protocol [24]. Briefly, the samples were loaded by SDS polyacrylamide gel electrophoresis (PAGE), and then the protein was transferred onto a polyvinylidene fluoride membrane using a wet transfer method. Membranes were blocked using 5% nonfat dry milk in PBS for 1 hour. After wash with TBST, the membrane was incubated with primary rabbit anti-Nrf2, rabbit anti-p65, or anti-GAPDH polyclonal antibody (1: 1000; Abcam) at 4°C overnight. After washing with TBST, the membrane was incubated with horseradish peroxidase conjugate secondary antibody (Abcam) at room temperature for 2 hours. The protein bands were visualized using an ECL chemiluminescence detection kit (Abcam).

### 2.5. DCFH-DA Assay

Dichlorodihydrofluorescein diacetate (DCFH-DA) assay was performed to detect intracellular ROS level as previously described [25]. Briefly, PC12 cells were inoculated into a 6-well plate and added with 20 nM DCFH-DA. Following incubation in a 37°C incubator for 24 hours, the cells was observed and photographed under an Olympus IX73 fluorescence microscope.

### 2.6. Mitochondrial Membrane Potential Assay

PC12 cells were inoculated into a 6-well plate at 1 × 10^5^ cells/mL with 2 mL cell suspension per well and incubated with 5 *μ*M rhodamine 123 (dissolved in dimethyl sulfoxide; Sigma-Aldrich) in 37°C for 45 minutes [26]. After washing, cells were collected by centrifugation at 1500 r/min for 5 minutes. Mean fluorescence intensity (MFI) was calculated using a flow cytometry.

### 2.7. SOD Activity and MDA Levels Measurement

Cells were lysed, and the supernatant was collected after centrifuged at 12000 *g* for 10 minutes. The intracellular SOD activity and MDA content were measured in strict accordance with the kit instructions (Jiancheng, Nanjing, China) [27].

### 2.8. Detection of Lactate Dehydrogenase (LDH) Activity

A*β*-induced cell injury was assessed using LDH activity assay according to manufacturer's instruction (Beyotime, China). Briefly, cell supernatant was incubated with reaction buffer and coenzyme I at 37°C for 15 minutes, followed by addition of 2,4-dinitrophenylhydrazine and incubation at 37°C for 15 minutes. After addition of 0.4 M NaOH and incubation for 5 minutes at room temperature, absorbance of cell supernatant at 450 nm was measured.

### 2.9. TUNEL Staining

A TUNEL apoptosis detection kit purchase from Beyotime was used to assess the cell apoptosis. PC12 cells were fixed using 4% paraformaldehyde at room temperature for 20 minutes followed by three times of wash with PBS for 5 minutes each time. After permeabilized with 1% Triton X-100, the cells were treated with 3% H_2_O_2_ for 10 minutes. After washing with PBS for three times, cells were incubated with TdT enzyme reaction solution containing TRITC-5-dUTP in the dark for 60 minutes. The nuclei were stained using 4′,6-diamidino-2-phenylindole (DAPI), and the fluorescence signal was visualized using a fluorescence microscope.

### 2.10. Statistical Analysis

All data are expressed as the mean ± SD. Analysis of variance and *q* test were used for comparison between groups. *P* < 0.05 was considered statistically significance.

## 3. Results

### 3.1. Maackiain Reduces A*β*_42_-Induced Cell Injury

**Figure 1:**
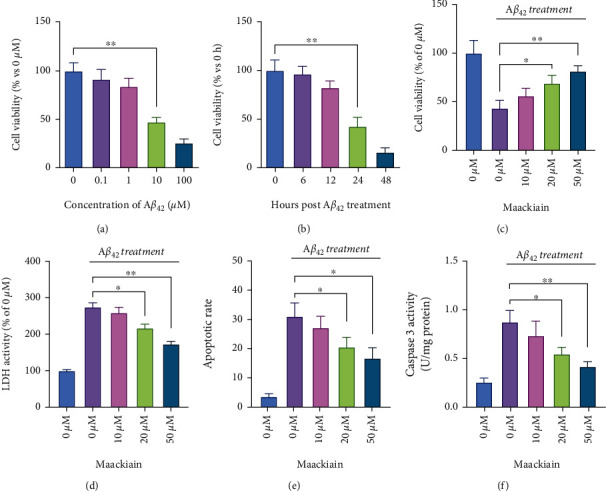
Maackiain prevented A*β*_42_-induced PC12 cell injury. (a) PC12 cells were stimulated by different doses of A*β*_42_ (0, 0.1, 10, and 100 *μ*M) for 24 hour or 10 *μ*M A*β*_42_ at different timepoints (0, 6, 12, 24, and 48 hours). CCK-8 assay was conducted to determine the cell viability in PC12 cells. (b) PC12 cells were stimulated by different doses of Maackiain (10, 20, and 50 *μ*M) for 6 hours and then exposed to A*β*_42_ (10 *μ*M) for additional 24 hours. CCK-8 (b) and LDH activity (c) assay were performed to detect the viability and injury severity in PC12 cells. TUNEL staining (d) and caspase-3 activity assay (e) were performed to assess the cell apoptosis. ∗*P* < 0.05 and ∗∗*P* < 0.01.

In order to determine the toxicity, different concentrations of A*β*_42_ were tested on PC12 cells using the CCK-8 assay. The results showed that a dose-dependent toxic effects of A*β*_42_ on PC12 cells and treatment with 10 *μ*M A*β*_42_ lead to reduction of cell viability to 50% of normal cells (Figure 1(a)). Further analysis showed 10 *μ*M A*β*_42_-induced toxic effects on PC12 cells time dependently with a significant effect for 24 hours (Figure 1(b)). Therefore, 10 *μ*M A*β*_42_ stimulation for 24 hours was selected as a condition to induce injury to PC12 cells for the following experiments. To determine the protective effects of Maackiain on cell injury induced by A*β*_42_, PC12 cells were pretreated with different doses of Maackiain (10, 20, and 50 *μ*M) six hours before 10 *μ*M A*β*_42_ stimulation. The results of CCk-8 and LDH activity assay showed that Maackiain prevented A*β*_42_-induced cell injury in a dose-dependent way (Figures 1(c) and 1(d)). The results of TUNEL staining demonstrated that A*β*_42_ induced apparent apoptosis in PC12 cells, which was remarkably reduced by treatment of Maackiain (Figure 1(e)). Moreover, Maackiain significantly inhibited A*β*_42_-induced caspase-3 activation in a concentration-dependent way (Figure 1(f)).

### 3.2. Maackiain Inhibits A*β*_42_-Induced Oxidative Stress in PC12 Cells

**Figure 2:**
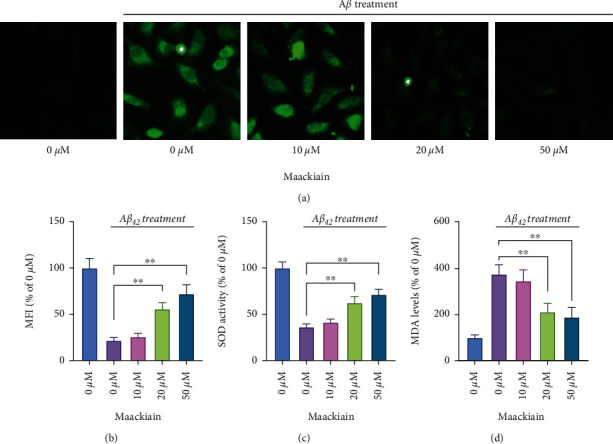
Maackiain abolished oxidative stress in PC12 cells stimulated with A*β*_42_. PC12 cells were stimulated by different doses of Maackiain (10, 20, and 50 *μ*M) for 6 hours and then exposed to 10 *μ*M A*β*_42_ for additional 24 hours, followed by determination of intracellular ROS level by DCFH-DA assay (a), mitochondrial membrane potential with rhodamine 123 coupled with flow cytometry (b), SOD activity (c), and MDA levels (d). ∗*P* < 0.05 and ∗∗*P* < 0.01.

To clarify mechanisms underlying the amelioration of A*β*_42_-induced cell injury by Maackiain in PC12 cells, DCFH-DA assay was performed to determine intracellular ROS level. A*β*_42_-induced ROS accumulation in PC12 cells as indicated by green fluorescence. Maackiain significantly reduced the generation of intracellular ROS by A*β*_42_ in PC12 cells dose dependently (Figure 2(a)). Flow cytometry analysis showed that A*β*_42_ decreased MMP in PC12 cells, and Maackiain restored intracellular MMP (Figure 2(b)). In addition, detection of intracellular SOD activity and MDA level revealed that Maackiain significantly abolished the decrease of SOD activity (Figure 2(c)) and increase of MDA levels induced by A*β*_42_ (Figure 2(d)).

### 3.3. Maackiain Prevents A*β*_42_-Induced Inflammatory Response

**Figure 3:**
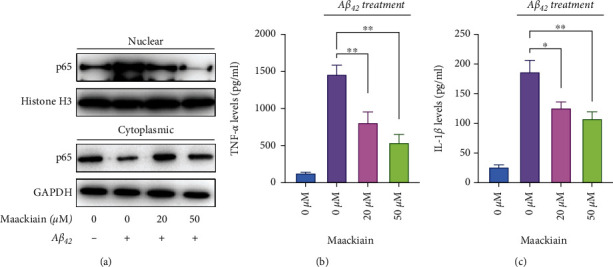
Inhibition of A*β*_42_-induced inflammatory response by Maackiain. PC12 cells were stimulated by different doses of Maackiain (10, 20, and 50 *μ*M) for 6 hours and then exposed to 10 *μ*M A*β*_42_ for additional 24 hours, followed by immunoblotting of the p65 levels in nuclear and cytoplasm (a). TNF-*α* (b) and IL-1*β* (c) levels in the supernatant were determined by ELISA. ∗*P* < 0.05 and ∗∗*P* < 0.01.

To investigate whether Maackiain affects A*β*_42_-induced inflammatory response, we detected NF-*κ*B activation in A*β*_42_-treated PC cells with or without Maackiain. The results showed that A*β*_42_ significantly promoted the translocation of p65, the key component of NF-*κ*B complex in PC12 cells, which was obviously prevented by Maackiain (Figure 3(a)). Consistently, ELISA detection of TNF-*α* and IL-1*β* protein level in the supernatant of PC12 cells indicated that Maackiain reduced the TNF-*α* (Figure 3(b)) and IL-1*β* (Figure 3(c)) levels that was increased by A*β*_42_.

### 3.4. Maackiain Promotes Nrf2 Nuclear Translocation *via* the PKC Signaling Pathway

**Figure 4:**
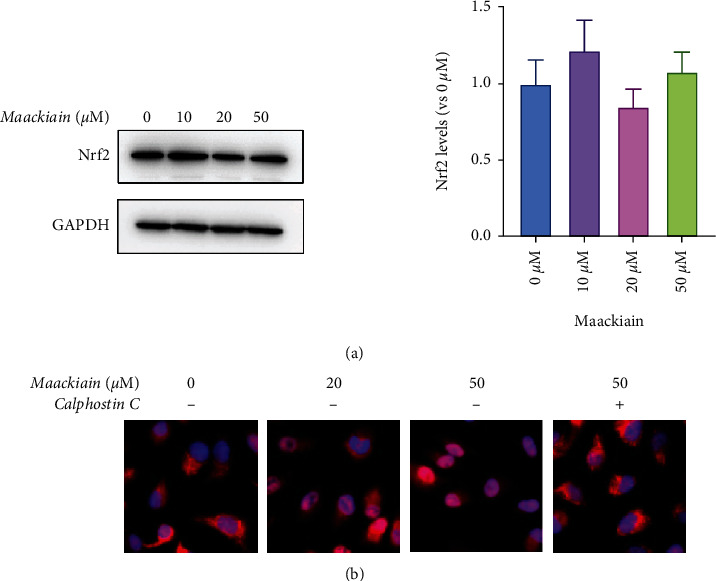
Maackiain promoted Nrf2 translocation dependent on PKC signaling. (a) PC12 cells were treated with different concentrations of Maackiain (0, 10, 20, and 50 *μ*M) for 12 hours. The levels of Nrf2 were determined using western blot analysis with GAPDH as loading control. (b) PC12 cells were treated with different concentrations of Maackiain (0, 10, 20, and 50 *μ*M) or calphostin C (100 nM) 1 hour before 50 *μ*M Maackiain stimulation. Immunofluorescence staining was performed to detect intracellular Nrf2 expression and location. Red fluorescence indicates Nrf2, and the nuclei were stained with DAPI (Blue).

We further determined Nrf2 expression in PC12 cells treated with Maackiain. The results of western blot analysis revealed that Maackiain did not affect Nrf2 expression (Figure 4(a)). The results of immunostaining showed that Nrf2 was present in the cytoplasm of PC12 cells, while Maackiain stimulation increased the intranuclear Nrf2 levels dose-dependently (Figure 4(b)), suggesting that Maackiain promotes the activation of Nrf2. After administration of PKC inhibitor calphostin C, the ability of Maackiain to promote intranuclear translocation of Nrf2 was weakened (Figure 4(b)).

### 3.5. Nrf2 Silencing or PKC Inhibition Attenuates the Neuroprotective Effects of Maackiain

**Figure 5:**
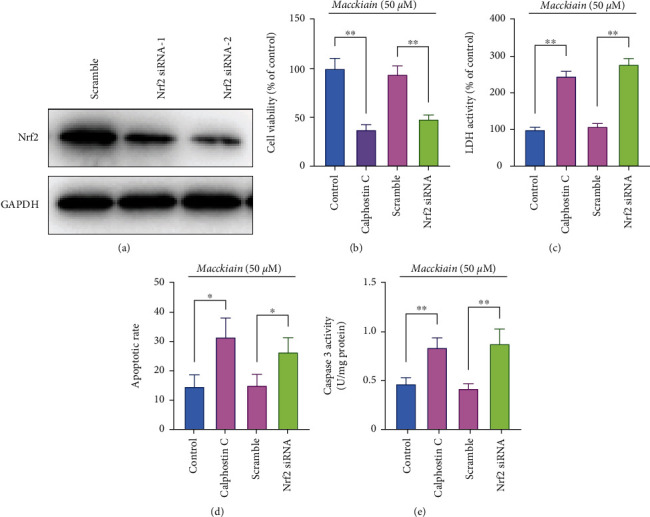
Effects of Nrf2 siRNA and calphostin C on PC12 cell injury. (a) Nrf2 and control siRNAs were transfected into PC12 cells. At 24 hours after transfection, the Nrf2 levels in PC12 cells were determined by immunoblotting. Prior to Maackiain (100 *μ*M) treatment, PC12 cells were transfected with Nrf2 siRNA or incubated with calphostin C for 1 hour and then exposed to A*β*_42._ CCK-8 (b) and LDH activity (c) assay were performed to detect cell viability and injury severity. Cell apoptosis was assessed by TUNEL staining (d) and caspase-3 activity assay (e). ∗*P* < 0.05 and ∗∗*P* < 0.01.

To validate the involvement of Nrf2 and PKC in the cytoprotective effects of Maackiain, prior to Maackiain treatment, PC12 cells were transfected with Nrf2 siRNA or treated with calphostin C, followed by exposure to A*β*_42._ As shown in Figure 5(a), transfected with Nrf2 siRNA significantly decreased the protein levels of Nrf2 in PC12 cells. CCk-8 assay showed that Nrf2 siRNA and calphostin C decreased the cell viability compared to that treated with Maackiain plus A*β*_42_ (Figure 5(b)), while LDH activity was increased compared to the control group (Figure 5(c)). TUNEL staining found that Nrf2 siRNA and calphostin C treatment increased PC12 cell apoptosis (Figure 5(d)) and promoted the caspase-3 activities in PC12 cells (Figure 5(e)).

### 3.6. Nrf2 Silencing or PKC Inhibition Attenuates the Antioxidant and Anti-inflammatory Effects of Maackiain

**Figure 6:**
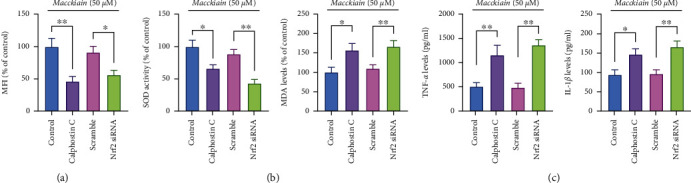
Effects of Nrf2 siRNA and calphostin C on oxidative stress. (a) Prior to treatment with 100 *μ*M Maackiain, PC12 cells were transfected with Nrf2 siRNA or incubated with calphostin C for 1 hour and then exposed to A*β*_42_. (a) MMP value was evaluated by rhodamine 123 staining. SOD activity and MDA levels (b) as well as TNF-*α* and IL-1*β* (c) were also assessed. ∗*P* < 0.05 and ∗∗*P* < 0.01.

To investigate the role of Nrf2 and PKC in Maackiain preventing against oxidative stress, prior to Maackiain treatment, PC12 cells were transfected with Nrf2 siRNA or incubated with calphostin C, followed by exposure to A*β*_42_. DCFH-DA assay was performed to detect intracellular ROS. After Nrf2 siRNA and calphostin C treatment, MFI value in the PC12 cells was weaker than that in the scramble and control group (Figure 6(a)), respectively. Nrf2 siRNA and calphostin C pretreatment decreased SOD activity and increased MDA levels (Figure 6(b)), as well as upregulated TNF-*α* and IL-1*β* (Figure 6(c)) in A*β*_42_-stimulated PC12 cells.

## 4. Discussion

Peony root is a perennial herb that is widely used in traditional Chinese medicine. Various active ingredients, flavonoids, saponins, and polysaccharides, have been identified from the extracts of peony root. Maackiain is an important flavonoid of peony root. Maackiain has been shown to alleviate adipogenic activity [28] and improve metabolic disturbance rats with diabetes mellitus [18]. Moreover, Maackiain shows a neuroprotective role in *Caenorhabditis elegans* with Parkinson's disease [22]. However, whether Maackiain can reduce A*β*_42_-induced neurotoxicity is unclarified. In this study, we showed that Maackiain reduced A*β*_42_-induced cell injury and apoptosis in PC12 cells. These findings suggest that Maackiain prevents A*β*_42_-induced neurotoxicity.

A*β*-induced oxidative stress plays an important role in the pathogenesis and development of AD [29]. Our results showed that Maackiain reduced ROS level as well as ΔΨ*m* in PC12 cells. Importantly, we found that Maackiain prevented A*β*_42_-induced decrease of ΔΨ*m* and SOD activity and increase of MDA content. The chronic inflammatory response of the nervous system is another important pathological feature of AD and plays a key role in promoting AD progression. In vivo and in vitro studies have demonstrated that abnormal deposition of A*β* in the brain is an initiation factor of neuroinflammatory response in AD [30]. A*β* promotes the release of inflammatory factors by binding to receptors on the surface of microglia and other neuronal cells. Moreover, A*β*-induced oxidative stress indirectly enhances the activation of inflammatory pathways. In this study, we found that A*β*_42_ significantly increased the mRNA levels and secretion of TNF-*α* and IL-1*β* in PC12 cells, which was however reduced by Maackiain pretreatment. These results demonstrate that Maackiain protects PC12 cells from A*β*_42_ through reducing A*β*_42_-induced oxidative stress and neuroinflammatory response.

Guo et al. [18] and Bai et al. [19] found that Maackiain exhibits antioxidative effect through promoting Nrf2 activation. Results from this study demonstrated that Maackiain did not affect Nrf2 expression but significantly increased intranuclear Nrf2 expression. These results indicate that Maackiain promotes intranuclear translocation of Nrf2, which is consistent with the findings of previous studies [18, 19]. In this study, we further investigated whether Nrf2 is involved in the antioxidative and anti-inflammatory effects of Maackiain. Our results showed that Nrf2 silencing using RNA interference technology, the antioxidative and anti-inflammatory effects of Maackiain were significantly weakened, and the protective effects of Maackiain on PC12 cells exposed to A*β* were also reduced. These findings suggest that promotion of Nrf2 nuclear translocation contributes to the antioxidative stress and anti-inflammatory effects of Maackiain.

Some protein kinases phosphorylate Nrf2 to alter its conformation and facilitate its separation from Keap1 [31]. PKC is a multifunctional serine/threonine kinase downstream of G protein-coupled receptor and is involved in various biochemical processes including the regulation of transcription factors [32, 33]. It has been reported that PKC can phosphorylate Nrf2 at Ser40 leading to Nrf2 dissociation from Keap1, entrance into the nucleus and promotion of antioxidant gene transcription [34, 35]. In this study, we showed that PKC-specific inhibitor calphostin C inhibited Maackiain in the intranuclear translocation of Nrf2. Moreover, calphostin C inhibited the effects of Maackiain on A*β*_42_-induced alteration of PC12 cell membrane potential, MDA content, and TNF-*α* and IL-1*β* levels and weakened the protective effects of Maackiain on cell viability.

Taken all together, our results demonstrated that Maackiain reduced A*β*_42_-induced oxidative stress, inflammatory responses, cell injury, and apoptosis in PC12 cells in a dose-dependent manner. Our results also revealed that Maackiain promoted Nrf2 intranuclear translocation through the PKC signaling pathway, and inhibiting PKC signaling pathway or knocking down Nrf2 weakened the antioxidative stress and anti-inflammatory effects of Maackiain. Our results suggest that Maackiain can prevent against A*β*_42_-induced neurotoxicity and holds promise to be used as a potential drug for AD treatment in the clinic.

## Data Availability

All data used to support the findings of this study are included within the article.
